# Supplementary material for: Naegleria amoebae seek confinement and crawl persistently through narrow spaces
Source: Proc Natl Acad Sci U S A. 2026 Sep 10;123(38):e2534165123. doi: 10.1073/pnas.2534165123 (PMC13598255; doi:10.1073/pnas.2534165123)
Supplement: Supplementary file 1 [file pnas.2534165123.sapp.pdf]

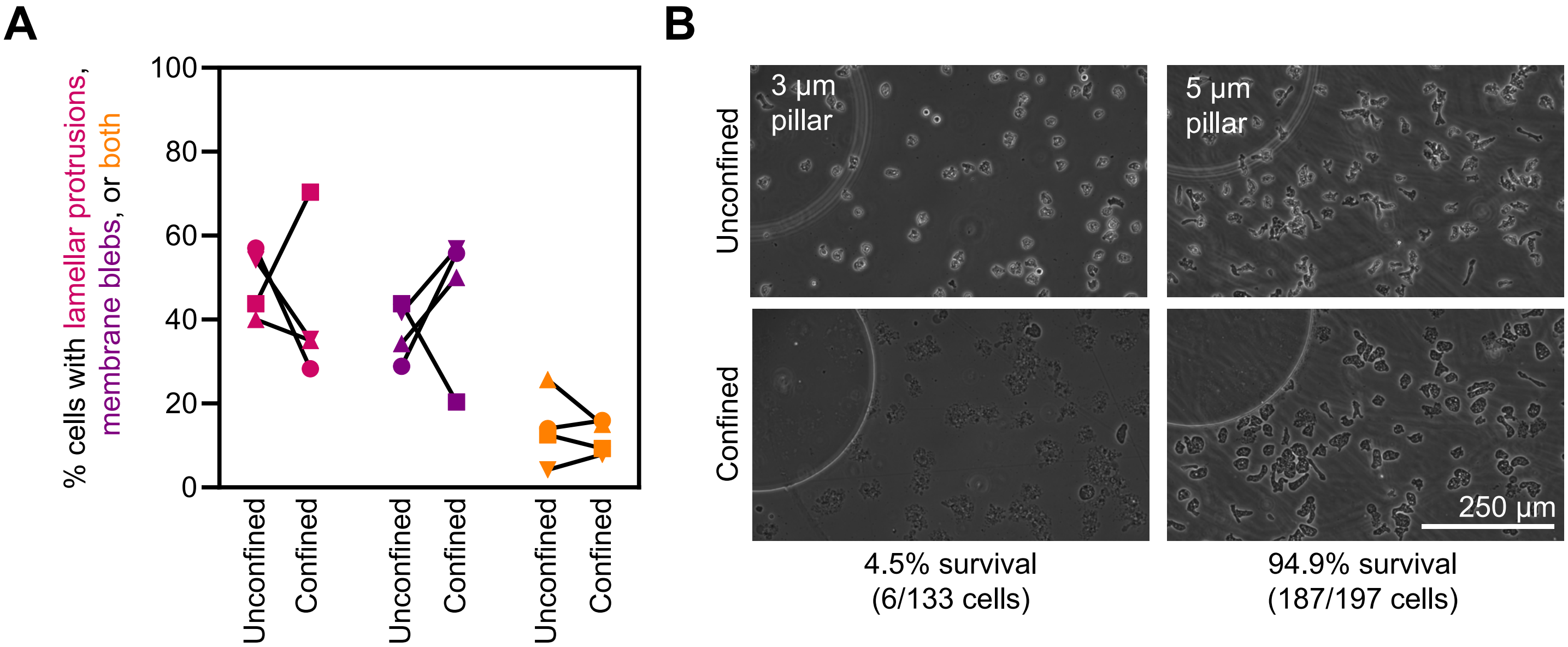

Figure S1. Confinement between coverslips leads to variable phenotypes. (A) The data from individual trials using confinement coverslips which were combined in Fig. 1C are shown by replicate (coordinated by shape). **(B)** 3 µm and 5 µm pillar sizes were tested to determine which size led to optimal confinement between coverslips. Confinement to 3 µm caused the vast majority of cells to burst. Images show the same representative area before and after confinement.

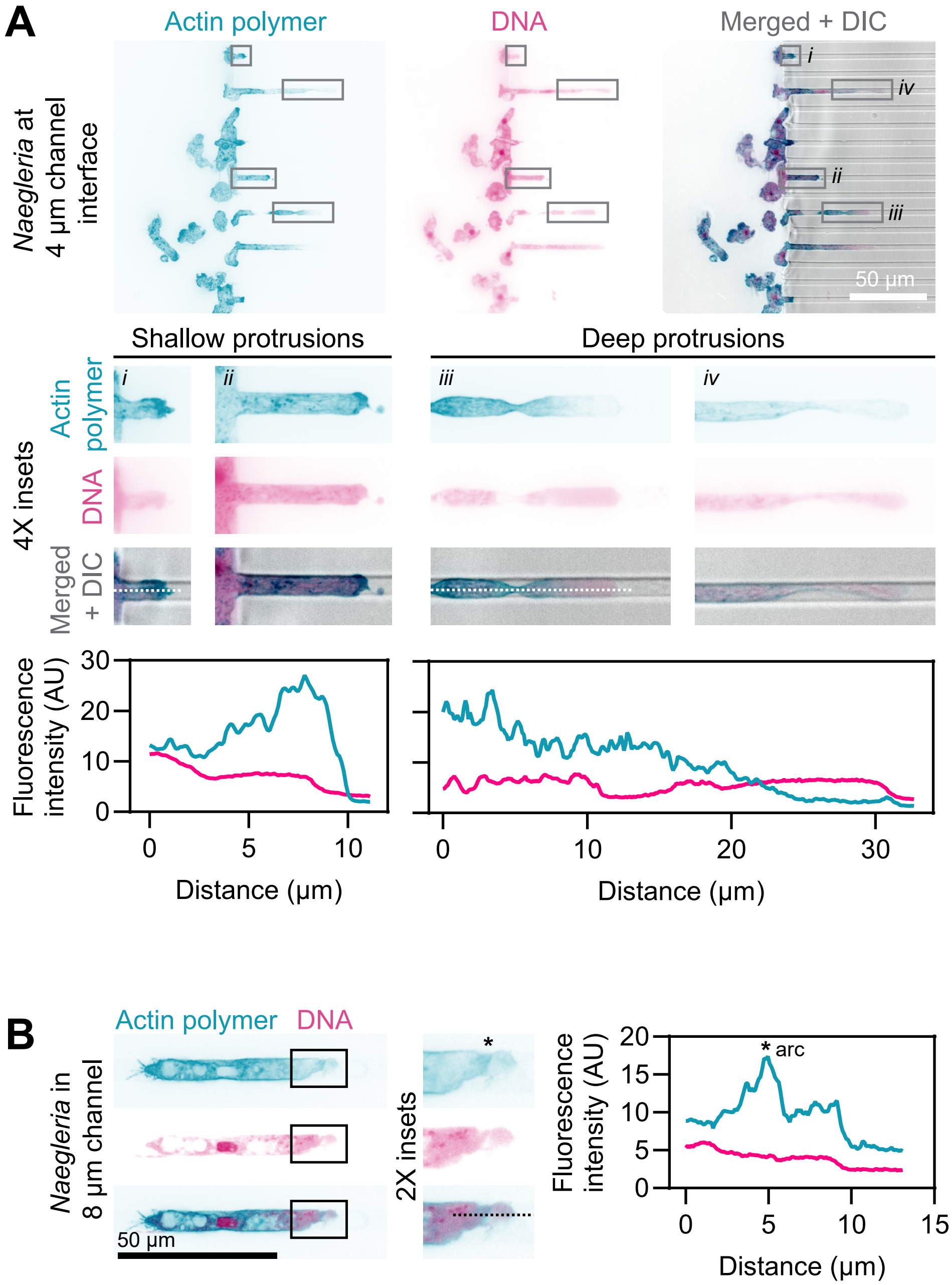

Figure S2. *Naegleria* cells switch from actin-rich protrusions to blebs when entering channels. (A) *N. gruberi* cells at the entrance of 4 µm channels were fixed and stained *in situ* for actin polymer (teal) using phalloidin and for DNA (magenta) using DAPI. Insets (middle row) show examples of shallow or deep protrusions. Linescans (bottom row) show actin polymer (teal) or DNA (magenta) staining intensity along the white dotted lines indicated in panels i and iii. (**B**) Cells were fixed and stained as in (A), but in an 8 µm channel. The leading edge of the cell is shown as an inset, with an actin arc indicated with an asterisk. A linescan along the black dotted line shows fluorescence intensity of actin polymer (teal) and DNA (magenta), with the asterisk corresponding to the position of the actin arc.

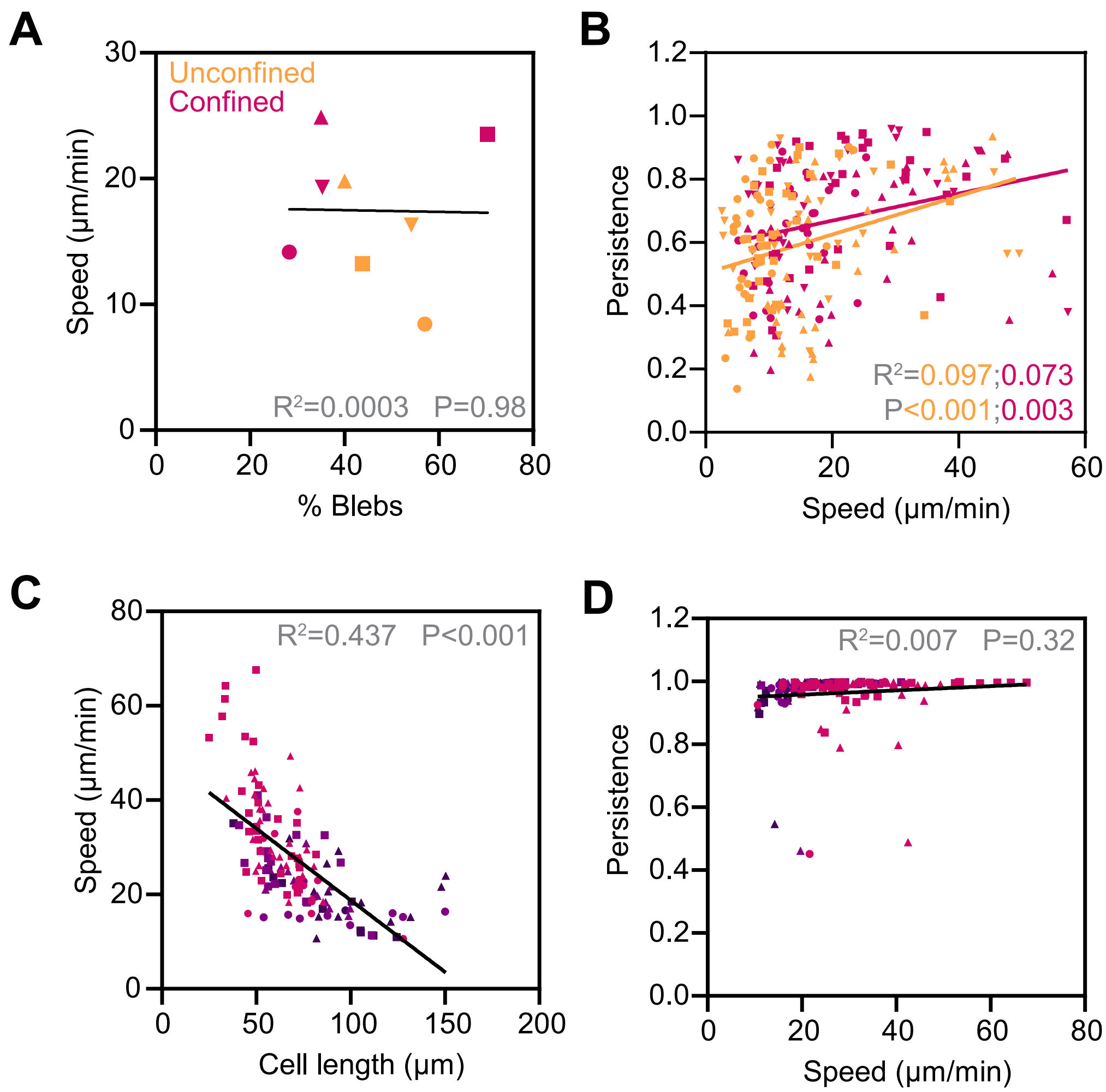

Figure S3. Cell speed is negatively correlated with cell length. (A) Data from experiments shown in Fig. 1C and Fig. 2B were plotted to compare average speeds and the percent of the cell population that used blebs. Data from unconfined populations (orange) and cells confined between coverslips (pink) were pooled for analysis. **(B)** Data from Fig. 2B-C were plotted to compare speed and persistence for each cell. Unconfined (orange) and confined (pink) populations were each analyzed separately. Symbol shapes are coordinated by trial and match A. **(C)** The lengths of cells from Fig. 2F in 8 µm channels (pink), 6 µm channels (light purple), or 4 µm channels were plotted against their speeds. **(D)** Data from Fig. 2F and 2H were plotted to compare speed and persistence for each cell. Data in all panels were analyzed by simple linear regression.

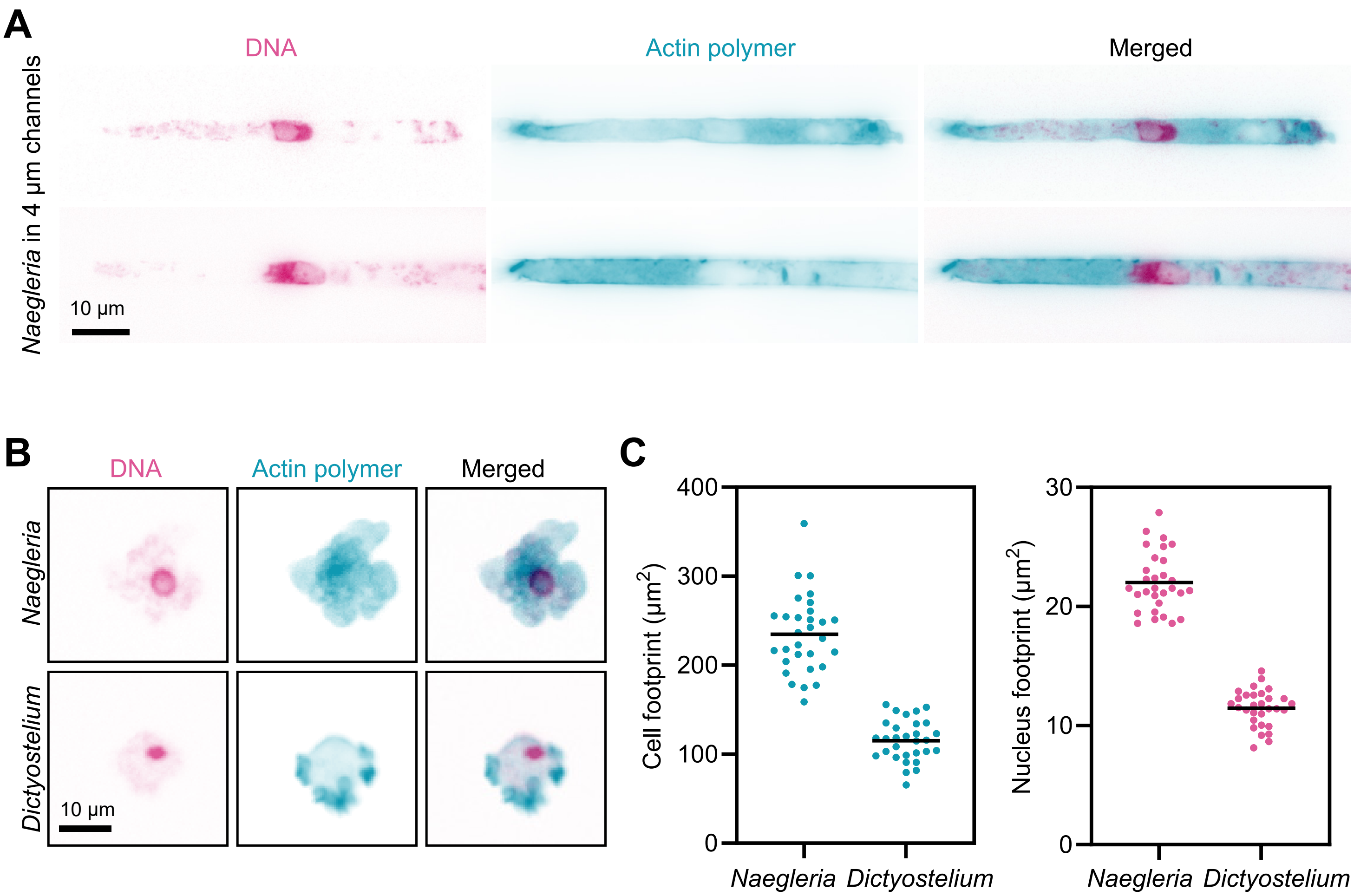

Figure S4. *Naegleria*'s nuclei deform in channels. (A) *N. gruberi* cells crawling in 4 µm channels were fixed and stained with Hoescht to visualize DNA (magenta), and phalloidin to label actin polymer (teal). Two representative cells with clear nuclear squishing are shown. **(B)** *N. gruberi* and *D. discoideum* cells were fixed then stained with DAPI to visualize DNA (magenta) and phalloidin to label actin polymer (teal). A representative cell for each species is shown. **(C)** Actin staining was used to outline the cell footprint to quantify cell areas, and DNA staining was used to quantify nuclear footprints. Each symbol represents an individual cell. The mean is indicated with a black line. N=30 cells.

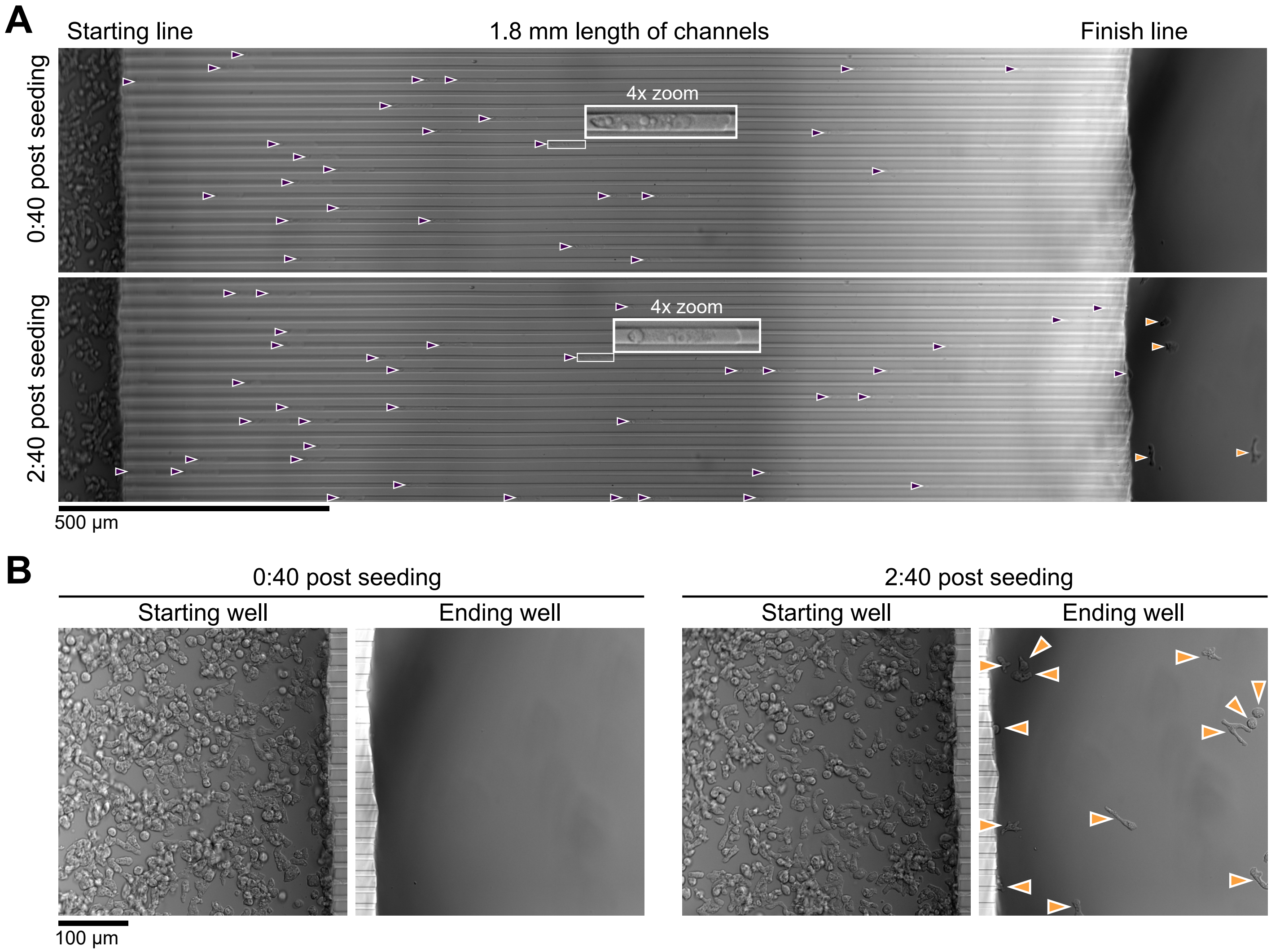

Figure S5. *Naegleria* amoebae can successfully complete a 1.8 mm dash. (A) *Naegleria* amoebae were seeded into a single well (left) of a dish with 8 µm channels, and imaged for 2 hours, starting 40 minutes post seeding. Images cover the full 1.8 mm run and the adjacent starting and ending wells. A single focal plane where the channels are in focus is shown for each timepoint. 4X insets show magnified amoebae from an optimized focal plane. Cells in channels are noted with purple arrowheads, cells that traversed the entire run length to reach the next well are shown with orange arrowheads. **(B)** Higher magnification views at optimized focal planes from the timepoints in A are shown for the starting well (where cells were seeded) and the ending well (where cells crawled). Cells that made it past the finish line are labeled with an orange arrowhead.

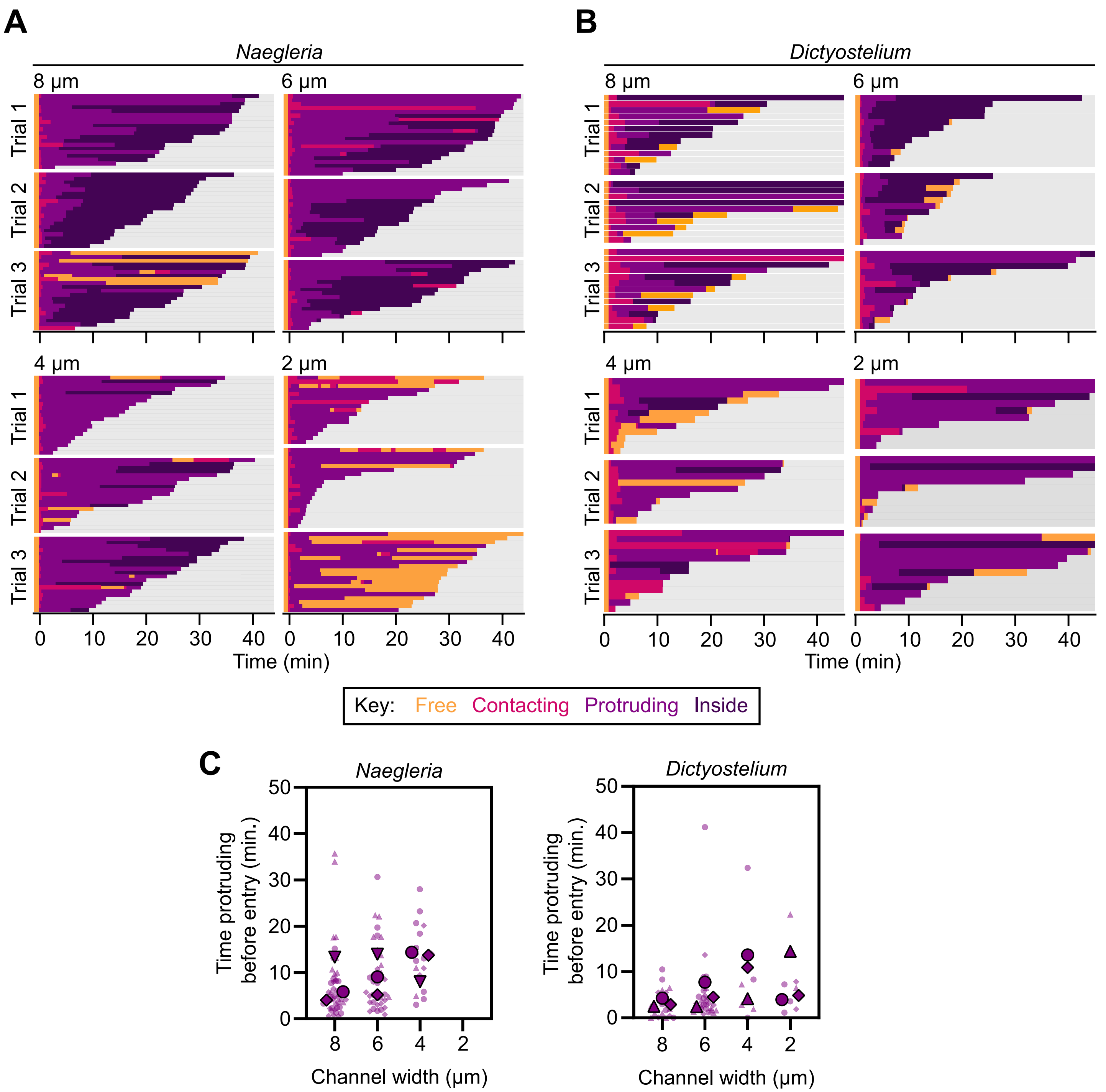

Figure S6. *Naegleria* enters microchannels at high rates. (A) *Naegleria*'s or **(B)** *Dictyostelium*'s interactions with 2-8 µm channels over 45 minutes of imaging are shown as horizontal bar plots, normalized such that t=0 represents the cell's first contact with the interface. Trial 1 for *Naegleria* is shown in Fig. 3B. **(C)** For each cell that entered a channel, the time spent protruding prior to entry was quantified and is shown as a SuperPlot. One way ANOVAs were performed on experiment level averages, and no significant differences were detected ($p>0.05$).

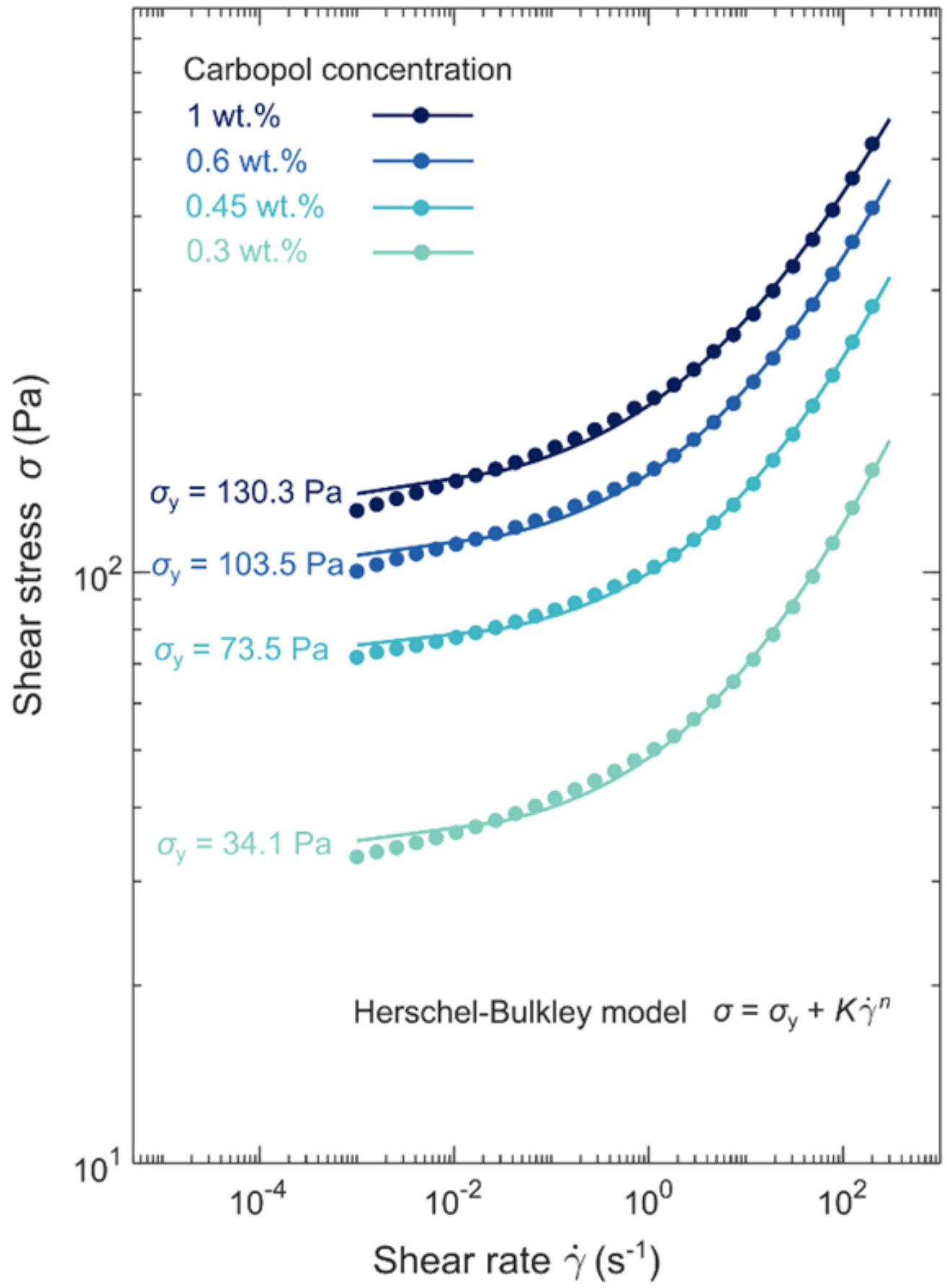

Figure S7. Rheological measurement of the Carbopol granular matrices. Shear stress of different Carbopol concentration versus unidirectional shear rates. The curves are fitted with the Herschel-Bulkley model for shear-thinning, viscoplastic fluids, where σ_y represents the yield stress, *K* is the consistency index, *n* is the flow index, and γ is the shear rate.

SUPPLEMENTAL MOVIE LEGENDS

Movie S1. Unconfined *Naegleria* form a range of blebs and lamellar protrusions. (A) The top cell shown in Fig. 1A forms lamellar protrusions to crawl. *In situ* fixing and staining shows these protrusions contain actin polymer. The scale bar indicates 10 μm. **(B)** The bottom cell from Fig. 1B forms blebs, which lack actin polymer. Flashing lines indicate actin arcs. The scale bar is 10 μm. **(C)** A cell forming a dynamic mixture of blebs and lamellar protrusions was imaged live, then fixed and stained *in situ*. Actin polymer staining shows an actin-rich lamellar protrusion towards the bottom of the cell, and an actin arc and cortex consistent with blebbing towards the top of the cell. **(D)** Two examples of cells that switch between blebs (labeled with a B) and lamellar protrusions (labeled with an L) are shown.

Movie S2. *Naegleria* amoebae favor interactions with surfaces and channels. Videos show cells interacting with 8, 6, 4, and 2 μm channels, and with a PDMS surface lacking channels. Data are from experiments shown in Fig. 3.

Movie S3. *Naegleria* use a mixture of blebs and lamellar protrusions to crawl in Carbopol. Two representative cells crawling through 0.45% Carbopol are shown, with a few examples of blebs (labeled B) and lamellar protrusions (labeled L). The first cell in the video is the same cell shown in Fig. 4A.
